# The reconstructive burden of inter-hospital transfers: resource utilization and clinical implications in tertiary trauma care

**DOI:** 10.3389/fsurg.2026.1766333

**Published:** 2026-05-15

**Authors:** Min Ji Kim, Hun Gil Cho, Il Jae Lee, Hyoseob Lim

**Affiliations:** Department of Plastic and Reconstructive Surgery, Ajou University School of Medicine, Suwon, South Korea

**Keywords:** patient transfer, reconstructive surgical procedures, referral and consultation, trauma centers, wound healing, soft tissue injuries, emergency medical services

## Abstract

**Introduction:**

Despite the presence of well-established trauma centers, many trauma patients are transferred due to unresolved wound complications. This often-overlooked issue highlights the critical need for specialized wound management and the essential role of plastic surgery (PS) in regional trauma centers. This study aims to emphasize the importance of effective wound management in trauma patients and examine the clinical outcomes of interhospital transfers.

**Methods:**

We performed a retrospective chart review of 92 trauma patients transferred to a regional trauma center from January 2020 to February 2022. We evaluated the injury severity score (ISS), initial resuscitation, and post-transfer treatment. To assess the clinical burden, resource utilization—including surgical intensity and length of stay (LOS)—was compared between the PS-involved group (*n* = 32) and the control group (*n* = 60).

**Results:**

Notably, 41.1% of patients were transferred from certified tertiary hospitals, indicating that even well-equipped facilities face challenges in wound management. Among the patients, 34.8% required specialized consultation with the PS department, with facial fractures (50%) and lower extremity skin defects (34.3%) being the most common indications. Compared to the control group, the PS-involved group required significantly higher surgical intensity (1.82 vs. 0.92, *p* < 0.001) and longer post-transfer hospitalizations (24.6 vs. 14.2 days, *p* = 0.002). These surgeries required approximately 2 operative debridements and 13 specialized wound care sessions per patient, underscoring the intensive care required for effective wound management.

**Conclusion:**

The increasing rate of inter-hospital transfers between tertiary centers underscores a critical need for specialized wound management. Our findings suggest that unresolved reconstructive needs are significant factors associated with these transfers, necessitating specialized plastic surgery involvement. Rather than a failure of management, these transfers may represent a proactive triage strategy for optimal care delivery. Integrating plastic surgery into trauma systems can optimize resource utilization and enhance the overall effectiveness of regional trauma care.

## Introduction

While trauma centers are designed to provide comprehensive care, a significant number of trauma patients still require interhospital transfers due to unresolved wound complications (such as extensive soft tissue defects, chronic infections, or exposed hardware requiring reconstructive surgery) (1, 2). These transfers indicate that even the most equipped facilities face challenges in managing complex wounds effectively. Wound management in trauma patients is often underestimated, yet it plays a critical role in patient recovery and outcomes (3). The necessity for specialized care is particularly evident in the management of complex soft tissue injuries, such as extensive skin defects, open fractures with exposed hardware, and progressive tissue necrosis. The plastic surgery (PS) department provide essential reconstructive expertise, including microsurgical tissue transfer and advanced debridement, which are critical for preventing secondary infections and ensuring stable wound coverage.

**Figure 1:**
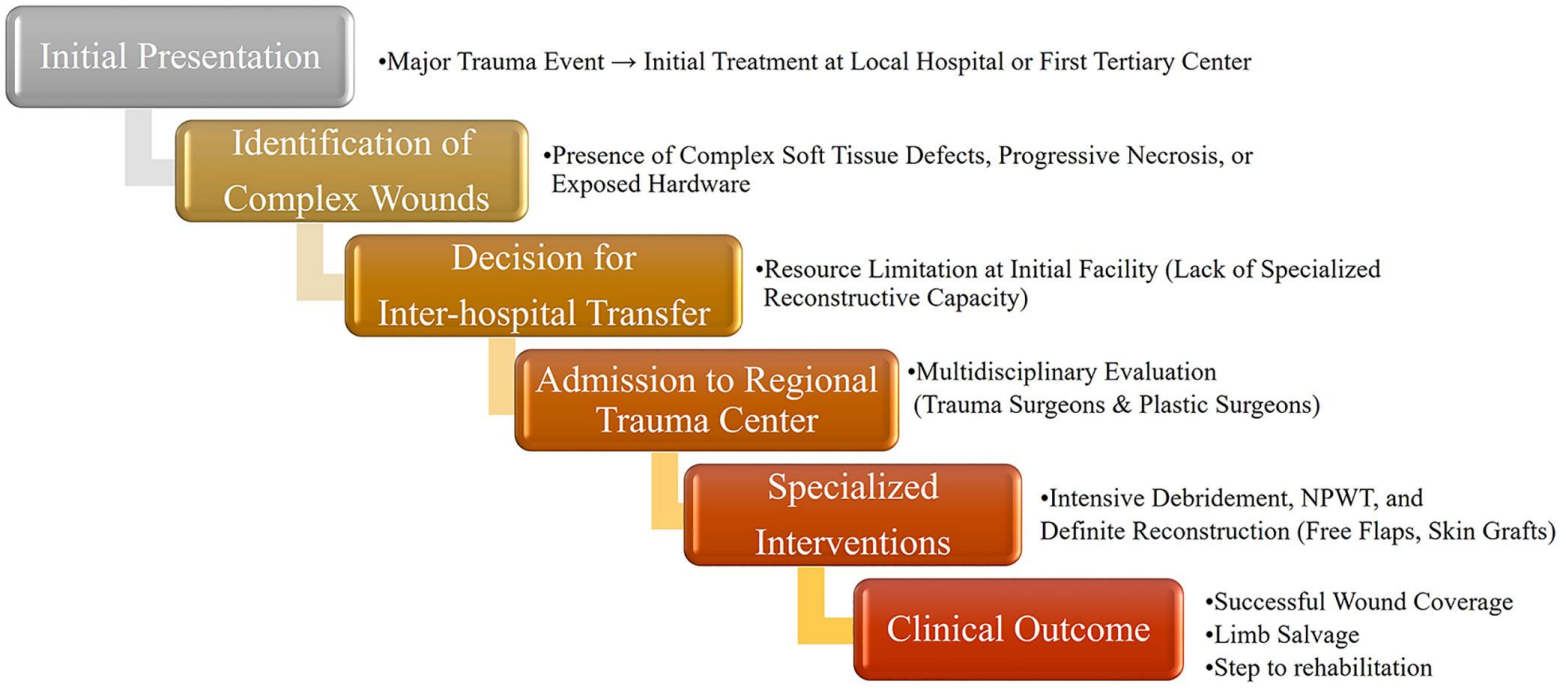
Clinical route map for specialized wound management in trauma care. This flowchart illustrates the systematic pathway from the initial major trauma event to definitive reconstruction at a regional trauma center. The route highlights the identification of complex wounds and the critical decision-making process for inter-hospital transfer when initial facilities face resource limitations in reconstructive capacity.

Major trauma patients have a high mortality rate, which is a key reason for the establishment of regional trauma centers aimed at reducing these deaths (4). However, this focus on reducing mortality has led to the neglect of other critical issues, such as effective wound management, that do not directly relate to vital signs but are nonetheless essential for optimal patient outcomes.The government has established a well-organized trauma system, leading to a decrease in preventable death rates. Preventable death rate has gained attention from the government, with the government initiating a well-organized trauma system in the country. After the establishment of a regional trauma center, preventable death rate decreased from 50.4% in 1997 to 19.9% in 2017 (5). However, systemic issues remain, particularly regarding the seamless integration of wound management in trauma care. The clinical pathway for trauma patients requiring specialized reconstructive services, from initial presentation to definitive care, is illustrated as a route map in Figure 1. Many patients are transferred between tertiary care centers due to the inability of initial facilities to adequately manage severe wounds, highlighting a critical gap in the trauma care continuum (6, 7).

This study aims to emphasize the importance of effective wound management in trauma patients and examine the clinical outcomes associated with interhospital transfers. By analyzing the role of plastic surgeons in these transfers, we seek to highlight the need for a robust and dynamic consultation system to improve patient outcomes and reduce unnecessary transfers.

## Methods

### Study design

This retrospective study was conducted at Regional Trauma Center, focusing on major trauma patients who were transferred from other hospitals between January 2020 and February 2022. The study aimed to evaluate the clinical outcomes of these patients, particularly focusing on wound management and the role of plastic surgeons.

### Patient selection

Patients included in the study were those transferred from other tertiary care centers, regardless of their Injury Severity Score (ISS). To ensure high data integrity, we focused on patients with complete clinical records regarding transfer indications and subsequent interventions. A total of 92 patients were identified. This cohort represents an exhaustive collection of a clinically rare and difficult-to-capture subset of trauma cases specifically transferred between tertiary care centers, rather than general trauma admissions. To analyze the impact of wound-related transfers, patients were categorized based on the interventions received to identify the subset of transfers driven by complex wound complications. This descriptive approach aims to analyze the specific clinical reasons and resource requirements of these inter-hospital transfers. The ‘PS involve’ group was defined as patients who required active plastic surgery intervention, which included at least one of the following: operative reconstructive procedures (e.g., skin grafting, flap surgery, or other reconstruction), bedside surgical debridement, or specialized continuous wound management using negative pressure wound therapy (NPWT). This objective definition ensures that the PS group represents patients with significant wound-related clinical needs, rather than those receiving only a single, non-interventional consultation. In contrast, the control group consisted of patients transferred for other clinical indications (e.g., neurological or orthopedic stabilization) who did not require plastic surgery involvement during their management. This classification allows for a direct comparison between wound-driven transfers and those motivated by other traumatic injuries. We defined debridement as procedures associated with surgical intervention for necrotic tissue under local or general anesthesia. Specialized wound care was defined as non-operative but advanced wound management required for wound bed preparation and infection control. This includes intensive interventions such as negative pressure wound therapy (NPWT) and therapeutic irrigation, which are distinct from routine bedside dressings.

### Data collection

Data were collected through a comprehensive review of medical records. The extracted information included demographic data such as age and sex, as well as injury details including the cause of trauma, initial ISS, and specific types of wounds. Additionally, we recorded pre-transfer treatments such as initial resuscitation, ICU care, and emergency operations. Post-transfer management data were also collected, focusing on consultations with the plastic surgery (PS) department, the number of debridements, and the frequency of specialized wound care sessions.

In the analysis of ISS, we categorized the score as >15 and <15. The ISS has been regarded as the gold standard in trauma severity and had prominence in trauma monitoring. While traumatic wounds in major trauma patients often present with extreme heterogeneity in depth and contamination, individual wound grading alone may not fully capture the systemic complexity of polytrauma cases. Therefore, we utilized the Injury Severity Score (ISS) as the primary objective metric for clinical urgency. As the validated gold standard in trauma surgery, ISS provides a reproducible and integrated measure of injury severity. This allowed for a standardized comparison of clinical status across different institutions, especially in the context of high-volume Level 1 Regional Trauma Centers where multi-organ stabilization and complex reconstructive needs must be addressed simultaneously. Moreover, several guidelines suggest that an ISS of 15 is the borderline that can determine whether a patient has a major trauma or not. This threshold was described as a predictive index of 10% mortality (8). Regarding referred institutions, tertiary general hospital defined as care of a technical and specialized nature for patients with severe and multiple trauma (9).

### Outcome measures

The primary objective of this study was to analyze the clinical burden and systemic determinants of inter-hospital transfers between tertiary trauma centers. To categorize the clinical status of these patients upon arrival, “stable” was defined as maintaining stable vital signs (systolic blood pressure > 90 mmHg and heart rate < 100 bpm) without immediate requirement for vasopressors or emergency blood transfusion. Additionally, “resuscitation” referred to active emergency stabilization efforts, such as intravenous fluid loading, blood product transfusion, or the use of inotropic agents during the initial care phase. While individual clinical outcomes are important, we focused on the reconstructive intensity (number of procedures and specialized care) to highlight the critical role of wound management in the regional trauma care system. Specifically, the primary outcome measures were defined as the frequency of operative debridements and specialized wound care sessions (including NPWT and irrigation), which serve as objective indicators of the reconstructive burden. Secondary outcomes included the length of ICU stay and overall hospital stay.

### Ethics approval and consent to participate

This study was approved by the Institutional Review Board (approval number: IRB-DB-2023-454) and was performed in accordance with the tenets of the Declaration of Helsinki. The needs of informed consents is waived due to its retrospective study nature.

### Statistical analyses

Descriptive statistics were used to summarize patient demographics and clinical characteristics. Comparisons between groups were made using the chi-square test for categorical variables and the t-test for continuous variables. To ensure the reliability of these systemic indicators, a complete case analysis was performed. Cases with missing primary data regarding transfer indications or treatment frequencies were excluded to maintain a high level of data integrity. A *p*-value of less than 0.05 was considered statistically significant. All analyses were performed using IBM SPSS Version 24.0 (IBM Corp., Armonk, NY, USA).

## Results

### Patient demographics and injury characteristics

**Figure 2:**
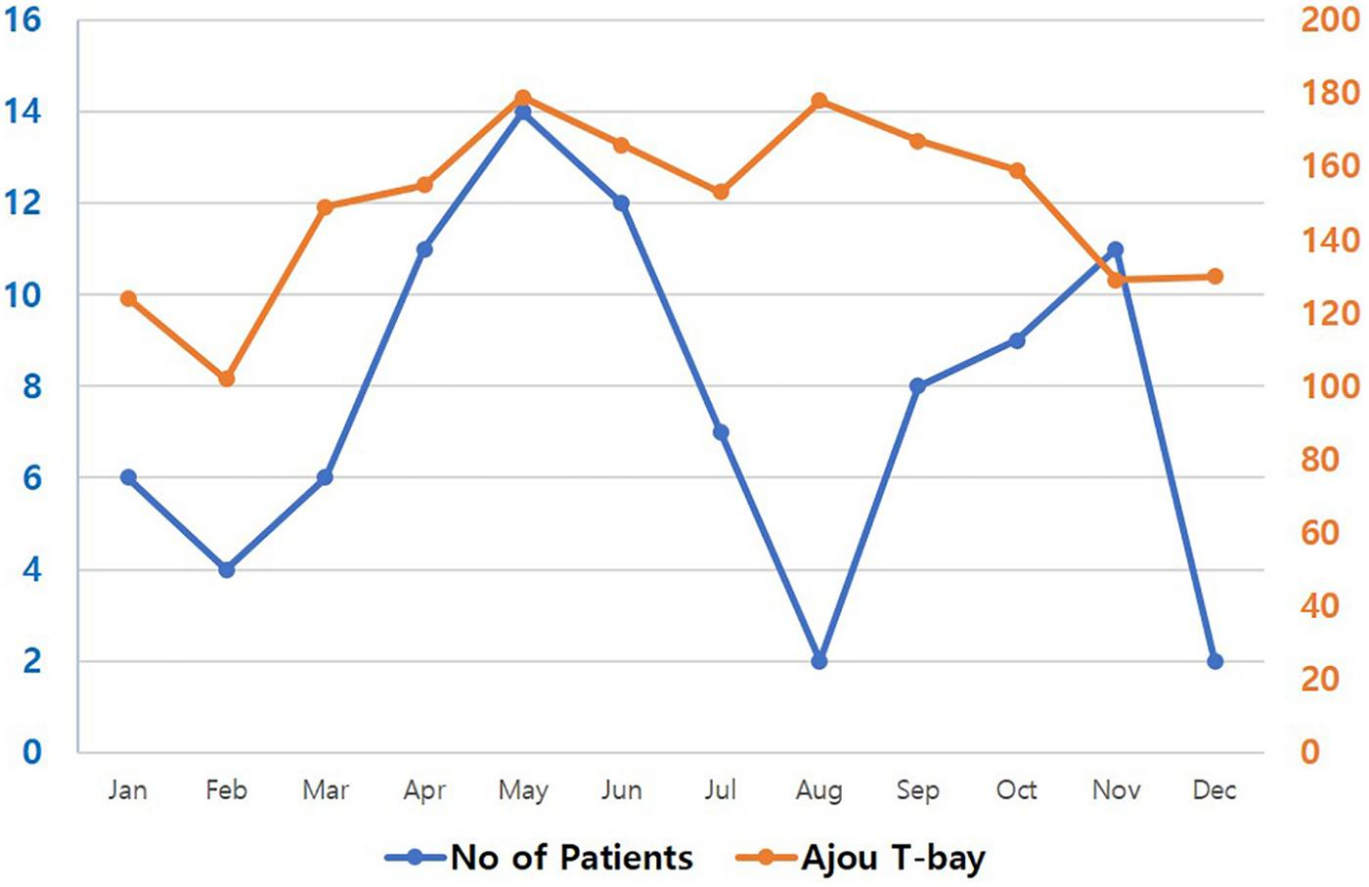
Monthly distribution of inter-hospital transfer. This figure illustrates the monthly distribution of patient transfers to Regional Trauma Center. The table displays the number of trauma patients transferred each month (blue dot). While the overall number of trauma patients at trauma center exhibits a similar trend to the total transfers, a notable decrease is observed in August and December, correlating with the center reaching its maximum transfer capacity during these months.

A total of 92 trauma patients transferred to Regional Trauma Center were included in the study. The monthly distribution of these inter-hospital transfers, which reflects the systemic burden and the center's seasonal capacity, is illustrated in Figure 2. Of these, 32 patients were involved in plastic surgery procedure associated patients (PS involve transfer group) and 60 were not involved (control group). In demographical analysis, there was no statistically significant difference between the two groups in terms of sex, arrival time, arrival route, cause of trauma, and causes of referral (Table 1). Regarding referred institutions, 65.20% were transferred from certified tertiary hospitals, indicating that even well-equipped facilities face challenges in wound management. Among the patients, 65.60% (21 patients) had initial open wounds, required consultation with the plastic surgery department.

**Table 1:** Comparative demographic analysis of PS-involved transferred patients and control.

Variables	Categories	*n* (%)	PS- involved transfer	Control	Total	*p*
Sex	Male	*n* (%)	28 (87.50)	44 (73.30)	72 (78.30)	0.184
	Female		4 (12.50)	16 (26.70)	20 (21.70)	
Arrival time	12:00–7:00	*n* (%)	7 (21.90)	6 (10.00)	13 (14.10)	0.135
	7:00–17:00		15 (46.90)	24 (40.00)	39 (42.40)	
	17:00–12:00		10 (31.30)	30 (50.00)	40 (43.50)	
Mode of arrival	Ambulance	*n* (%)	30 (93.80)	52 (86.70)	82 (89.10)	0.619
	Car		2 (6.30)	6 (10.00)	8 (8.70)	
	Airline		0 (0.00)	2 (3.30)	2 (2.20)	
Causes of trauma	Traffic accident	*n* (%)	16 (50.00)	27 (45.00)	43 (46.70)	0.794
	Industrial accident		9 (28.10)	16 (26.70)	25 (27.20)	
	Violence		7 (21.90)	17 (28.30)	24 (26.10)	
Referred institution	General hospital	*n* (%)	11 (34.40)	21 (35.00)	32 (34.80)	0.004*
	Tertiary hospital		21 (65.60)	39 (65.00)	60 (65.20)	
Causes of referral	Request of guardian	*n* (%)	21 (65.60)	46 (76.70)	67 (72.80)	0.156
	Professional trauma treatment		5 (15.60)	11 (18.30)	16 (17.40)	
	Impossibility of emergency treatment		5 (15.60)	3 (5.00)	8 (8.70)	
	Lack of ICU bed		1(3.10)	0(0.00)	1(1.10)	

PS, plastic surgery; ICU, intensive care unit.

*p < 0.05.

### Pre-transfer and post-transfer treatments

Regarding pre-transfer treatments, there was a statistically significant difference in the clinical severity between the two groups; specifically, the PS-involved group showed a higher trend of intensive interventions than the control group, including resuscitation (16.3%), ICU care (18.5%), and emergency operations (17.4%) (*p* = 0.047) (Table 2). Post-transfer, the majority of the patients required significant medical interventions. In terms of initial consultations with other departments (excluding plastic surgery), the PS-involved group had a significantly lower rate of neurosurgery or orthopedic surgery consultations compared to the control group (*p* = 0.001). In post-transfer stage, there was no significant difference in emergency operation after transfer and post-transfer ICU care.

**Table 2:** At level I trauma center arrival, comparative analysis of initial management.

Variables	Categories	*n* (%)	PS involve transfer	Control	Total	*p*
At arrival vital sign	Stable	*n* (%)	30 (93.80)	56 (93.30)	86 (93.50)	0.999
	Unstable		2 (6.30)	4 (6.70)	6 (6.50)	
Pre_Transfer treatment	Resuscitation	*n* (%)	4 (12.50)	11 (18.30)	15 (16.30)	0.471
	No		28 (87.50)	49 (81.70)	77 (83.70)	
Pre_Transfer treatment	ICU care	*n* (%)	3 (9.40)	14 (23.30)	17 (18.50)	0.100
	No		29 (90.60)	46 (76.70)	75 (81.50)	
Pre_Transfer treatment	Emergency operation	*n* (%)	9 (28.10)	7 (11.70)	16 (17.40)	0.047*
	No		23 (71.90)	53 (88.30)	76 (82.60)	
Initial consultation (Excluding plastic surgery)	NS	*n* (%)	9 (28.10)	14 (23.30)	23 (25.00)	0.001**
	OS		13 (40.60)	40 (66.70)	53 (57.60)	
	Others		3 (9.40)	6 (10.00)	9 (9.80)	
Emergency operation	Yes	*n* (%)	14 (43.80)	22 (36.70)	36 (39.10)	0.507
	No		18 (56.30)	38 (63.30)	56 (60.90)	
Post transfer ICU care	Yes	*n* (%)	13 (40.60)	23 (38.30)	36 (39.10)	0.827
	No		19(59.40)	37(61.70)	56(60.90)	

PS, plastic surgery; NS, neuro surgery; OS, orthopedic surgery.

*p < 0.05.

### Injury severity score (ISS) analysis

Patients were categorized based on their ISS, ISS analysis showed that in PS involve patients, 68.75% had a score of >15 and 31.25% had a score of <15 (Table 3). This means that the PS involve transfer group had a higher proportion of major trauma patients than did the control group (68.75% vs. 46.67%. *p* = 0.012). Although ISS may serve as a potential confounder in the analysis, this baseline difference is also likely to reflect the clinical nature of the injuries themselves, as major soft tissue injuries and complex facial traumas are directly scored under the AIS categories. According to the AIS (Abbreviated Injury Scale) guidelines, major soft tissue injuries and complex facial traumas which characterize our PS group (e.g., 50.0% facial injuries and 53.7% skin defects) are directly scored under the ‘External’ and ‘Face’ categories. These high AIS scores naturally elevate the total ISS, justifying why patients requiring specialized reconstruction inherently present with higher severity scores. Among those referred to the plastic surgery department, a significant portion had severe injuries, emphasizing the complexity of cases managed.

**Table 3:** A relationship between wound problems and transfer.

Variable	PS-involved transfer (*n* = 32)	Control (*n* = 60)	*p*-value
Injury Severity Score (ISS)			0.012*
ISS ≥ 15 (Major Trauma), n(%)	22 (68.75)	28 (46.67)	
ISS < 15 (Minor Trauma), n(%)	10 (31.25)	32 (53.33)	
Total	32 (100.00)	60 (100.00)	

PS, plastic surgery; ISS, injury severity score;.

*P*-values refer to the comparison between the PS-involved and control groups.

### Plastic surgery interventions

**Figure 3:**
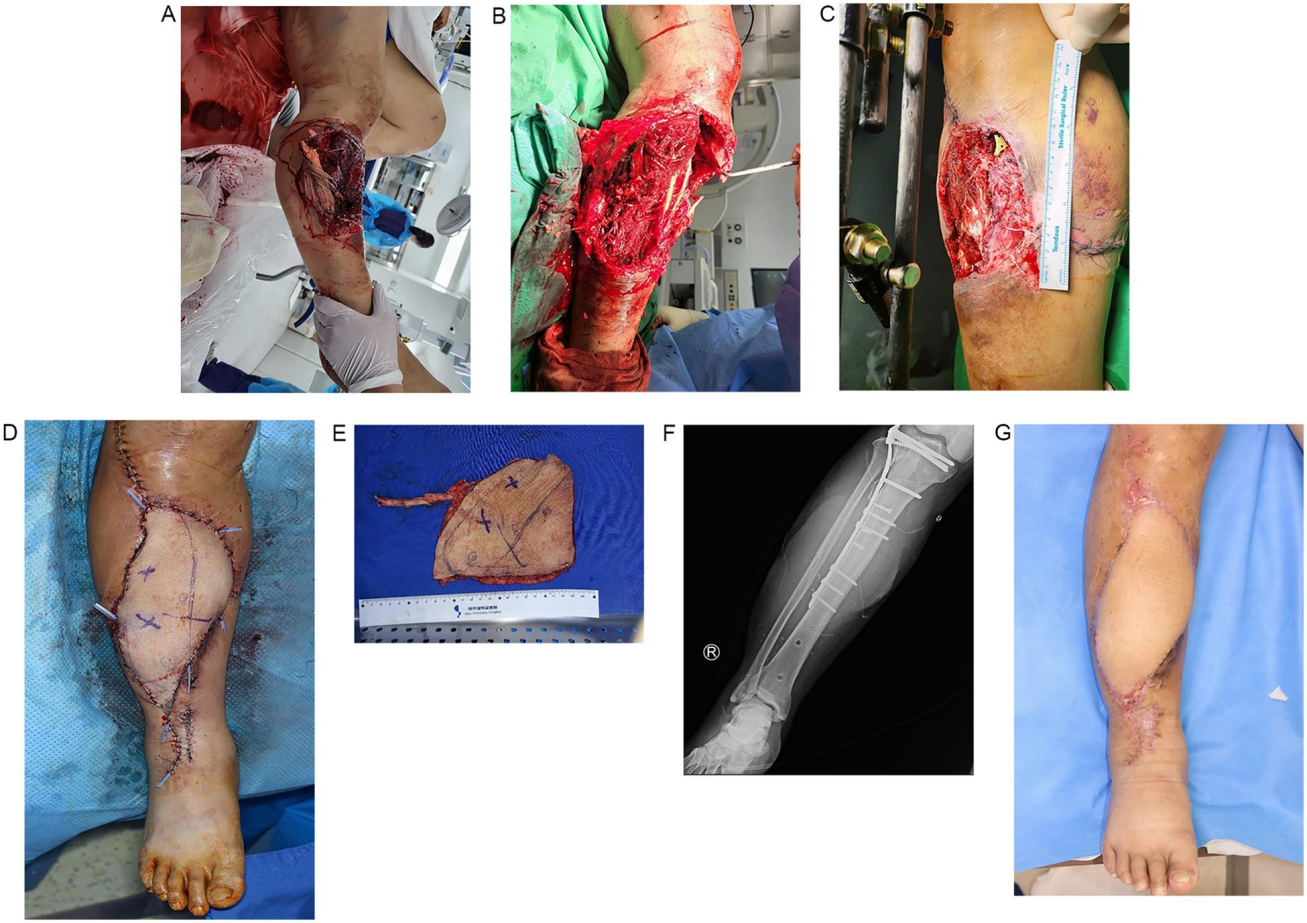
Representative case of a tertiary to tertiary hospital transfer. **(A)** Initial presentation of a 41-year-old male patient with a severe open tibial shaft fracture and extensive soft tissue injury following a motorcycle accident. The ischemic appearance of the muscle in the initial photograph indicates potential necrosis and impending infection. **(B)** Emergency external fixation and multiple debridements were performed at the first tertiary hospital, yet the patient's condition worsened due to progressive muscle necrosis and systemic rhabdomyolysis. **(C)** Due to the high septic state and extensive soft tissue defect, the patient was transferred to our better-equipped tertiary center. Here, a specialized plastic surgeon managed the wound coverage and infection control, supported by a trauma surgeon who stabilized the patient's general condition. **(D)** Final reconstruction involved the application of an anterolateral thigh free flap for soft tissue coverage, with the orthopedic fixation revised to intramedullary nailing. **(E)** Intraoperative image of the flap harvest. **(F)** Postoperative status showing tibial alignment. **(G)** Final postoperative condition with the extremity well reconstructed and the patient prepared for discharge, demonstrating full ambulation.

Plastic surgery interventions were crucial for the management of complex wounds. The standardized mapped route for these interventions, including multidisciplinary evaluation and intensive debridement, is further detailed in Figure 1. A representative case of a tertiary-to-tertiary hospital transfer, managed successfully through the specialized reconstructive route mapped in Figure 1, is presented in Figure 3. This case specifically demonstrates a common clinical scenario where initial orthopedic internal fixation (ORIF) is performed, yet definitive wound management remains unaddressed due to a lack of specialized reconstructive resources at the primary facility. Figure 3 highlights that while skeletal stability is frequently achievable in most tertiary centers, the absence of plastic surgery expertise often necessitates inter-hospital transfer for essential soft tissue reconstruction and limb salvage. Regarding the 32 patients who underwent post-referral treatment in the plastic surgery department, facial fracture and lower extremity skin defect were most common wounds (Table 4). If one patient underwent multiple surgeries, the number of surgeries was counted twice. On average, patients who required plastic surgery had approximately 2 operative debridements and 13 specialized wound care sessions (mean 1.76 and 12.75, respectively). The high frequency of these interventions serves as an objective surrogate for wound complexity and the intensive resource utilization required for successful limb salvage and infection control in these severe cases. These interventions highlight the intensive care required for effective wound management in trauma patients.

**Table 4:** An analysis on post plastic surgery department referral treatment.

Variables	Category	*n*	%
Wound site	Face	16	50.0
	Lower extremity	11	34.3
	Trunk	1	3.1
	Upper extremity	4	12.5
Diagnosis	Fracture	11	26.19
	Skin defect	22	53.66
	others	8	19.51
Operation	ORIF	11	36.8
	Skin graft	6	26.3
	Local flap	4	15.8
	Free flap	4	15.8
	others	9	21.1
Number of operative debridements (mean ± SD)	1.76 ± 0.46		
Number of specialized wound care sessions (mean ± SD)*	12.75 ± 3.45		

ORIF, open reduction and internal fixation; SD, standard deviation.

*Specialized wound care includes non-operative interventions such as negative pressure wound therapy (NPWT) and therapeutic irrigation.

### Comparative resource utilization

A comparative analysis of clinical resources showed that the PS-involved group required significantly higher surgical intensity (1.82 vs. 0.92, *p* < 0.001) and longer post-transfer hospital stays (24.6 vs. 14.2 days, *p* = 0.002) compared to the control group (Table 5). The markedly higher NPWT application rate (71.9% vs. 11.7%, *p* < 0.001) in the PS-involved group further underscores the specialized and resource-intensive nature of care required for these complex reconstructive cases. To identify independent predictors of surgical intensity, a multivariable linear regression analysis was performed. As shown in Table 6, PS involvement was significantly associated with higher surgical intensity (*β* = 0.84, *p* < 0.001), independent of injury severity (ISS) and pre-transfer surgical history.

**Table 5:** Comparative analysis of clinical resource utilization and operational efficiency.

Variable	PS-involved Group (*n* = 32)	Control Group (*n* = 60)	*p*-value
Surgical Intensity			
Total Surgical Interventions (mean ± SD)	1.82 ± 0.64	0.92 ± 0.35	< 0.001^c^
Mean Debridements (All patients)^a^	0.69 ± 1.03	0.25 ± 1.07	0.061
Mean Debridements (Operated only)^b^	1.76 ± 0.46	-	-
Advanced Wound Management			
NPWT Application Rate (%)	71.9% (23/32)	11.7% (7/60)	< 0.001^c^
Specialized Wound Care Sessions (mean ± SD)	4.75 ± 10.00	1.72 ± 9.21	0.148
Operational Efficiency & Resource Use			
Time to PS Consultation (hours)	6.4 ± 4.2	-	-
Post-transfer Length of Stay (days)	24.6 ± 12.3	14.2 ± 8.1	0.002^c^
Post-transfer ICU Care Rate (%)	40.6% (13/32)	38.3% (23/60)	1.000

PS, plastic surgery; NPWT, negative pressure wound therapy; ICU, intensive care unit; SD, standard deviation.

a Calculated across the entire cohort (*n* = 32 vs. *n* = 60) to compare systemic resource burden.

b Calculated only for patients who underwent operative debridement (*n* = 13) as detailed in Table 4.

c Statistically significant difference (*p* < 0.05).

**Table 6:** Multivariable linear regression analysis for factors associated with surgical intensity.

Variable	Coefficient (*β*)	Standard Error	*p*-value
PS Involvement (Yes vs. No)	0.84	0.12	<0.001*
Injury Severity Score (ISS)	0.02	0.01	0.082
Pre-transfer Emergency Operation (Yes vs. No)	0.15	0.14	0.284

PS, plastic surgery; ISS, injury severity score. The model adjusts for systemic injury severity and prior surgical history to identify independent predictors of total surgical interventions.

*p < 0.05.

## Discussion

This study underscores the frequent necessity of interhospital transfers among major trauma patients, suggesting a strong association between unresolved wound management and the need for transfer, even between well-established tertiary care centers. In trauma patients, transfer is a crucial issue, often determining the difference between successful recovery and deterioration (10, 11). Despite advances in trauma care and the availability of comprehensive facilities, the management of complex wounds remains a significant factor that may necessitate transfer when local institutional resources are exhausted. Wound care is a fundamental aspect of trauma treatment that is often overlooked, yet it is crucial for the recovery and long-term health of patients (12, 13). Our findings suggest that even tertiary hospitals face challenges in providing the highly specialized reconstructive care required for complex cases, which appears to be associated with the frequent transfer of patients to regional trauma centers. Although this represents a specific subset of patients, these cases serve as a critical indicator of underlying systemic resource gaps in reconstructive capacity that can exist even within high-level medical institutions. The conclusions of this study are in line with those found in the previous other research which emphasizes the crucial role of plastic surgery in managing complex wounds and improving patient outcomes, reflecting a common direction with prior research on the subject (14–16).

As previously noted, we confirmed that tertiary to tertiary hospital transfers accounted for a large proportion of all transfer patients, and why this happened was the basis of our study. A key observation in our analysis was the significantly higher ISS in the PS-involved group compared to the control group (68.75% vs. 46.67%, *p* = 0.012). While this difference represents a potential confounder that was adjusted for in the analysis, it may also reflect the clinical complexity of the injuries themselves, given that extensive soft tissue defects and complex facial traumas directly contribute to elevated ISS values. According to the AIS guidelines, the complex facial traumas and extensive soft tissue defects that characterize the PS-involved group directly contribute to elevated ISS values. Therefore, the increased frequency of interventions and the necessity for transfer in this group are likely associated with the inherent complexity of these injuries, which present both as high systemic severity (high ISS) and as specialized reconstructive challenges. These baseline differences, particularly the higher frequency of pre-transfer emergency operations in the PS-involved group, while representing potential confounders, may also reflect system-level referral patterns where unresolved complex reconstructive needs appear to be associated with the necessity for transfers between tertiary care centers. Rather, they reflect system-level referral patterns where unresolved complex reconstructive needs appear to be associated with the necessity for transfers between tertiary care centers. Patients who transferred from another hospital usually proceed to the next steps of treatment, most of which are emergency operations. In our analysis of post-transfer procedures, the PS involve transfer group had a higher proportion of emergency operations than did the control group (43.80% vs. 36.70%), implying that that PS involve problem was not controlled at the pre-transfer stage even though many of patients already underwent life-threatening emergency operations before transfer. Therefore, a significantly higher proportion of transfer cases in the PS-involved group appear to be associated with unresolved wound complications. In addition, our study showed that in the PS involve transfer group, more patients were transferred from a tertiary hospital than from a second general hospital (65.60% vs. 34.40%). This further shows that there were many PS involvements among problems that were not resolved, even in the relatively well-organized tertiary hospital. Therefore, the reason why tertiary to tertiary interhospital transfer is the complicated wound problem, that cannot be overlooked as a reason. In this context, inter-hospital transfer should be viewed as a proactive triage mechanism to ensure optimal care delivery rather than a failure of initial management. Recent literature suggests that for patients requiring complex reconstruction, timely transfer to specialized centers is associated with favorable clinical outcomes and increased likelihood of discharge home (21). Therefore, our findings highlight both the systemic resource gaps and the clinical necessity of early identification and referral to facilities with integrated reconstructive expertise.

Why does a wound problem that cannot be treated in a tertiary hospital with relatively necessary professionals and equipment occur? This discrepancy may reflect a systemic oversight in trauma policy, which predominantly prioritizes immediate vital sign stabilization and skeletal fixation while overlooking the critical necessity of soft tissue management. In high-severity cases, such as comminuted fractures, extensive soft tissue damage is an inevitable accompaniment; however, many facilities accept these patients without a viable strategy for specialized wound care. This “treatment without a plan” for complex wounds may contribute to clinical stagnation at the primary facility, and inter-hospital transfer tends to follow once reconstructive options become limited. Previous studies have primarily focused on the initial treatment and life-saving measures for trauma patients, often neglecting the importance of sustained wound management. However, our study emphasizes the crucial role of wound care in the overall management of trauma patients. The necessity of plastic surgery consultations and interventions for a significant portion of transferred patients highlights the importance of specialized wound management (17). This finding differentiates our study from prior research and underscores the critical need for integrating advanced wound care protocols into the standard trauma care practices (16, 18). In the present study, the average number of debridement was 1.75 and that of specialized wound care was 12.75. Similarly, the majority of workload for wound management was found to be related to soft tissue defect caused by trauma and injuries requiring microsurgery in a high-level trauma center. The role of plastic surgeons in providing specialized care for complex wounds is pivotal, and their involvement is essential for improving patient outcomes and reducing the need for further transfers (19, 20).

It is important to emphasize that our analysis originated from an investigation into why inter-hospital transfers occur between tertiary care centers, rather than being a study focused solely on plastic surgery outcomes. Our findings reveal that a significant proportion of these high-level transfers appear to be associated with unresolved wound complications and reconstructive gaps. The categorization into a PS-involved group was therefore an analytical step to objectively characterize the clinical reality and the intensive resource requirements of these specific cases.

While this study provides valuable insights, it is not without limitations. First, as a retrospective analysis, there is a potential for indication bias and selection bias; the decision to involve plastic surgery was made by the treating trauma surgeons based on clinical judgment, which may have led to a non-objective definition of the PS-involved group. Second, the study was conducted at a single institution, which may limit the generalizability of the findings. Specifically, this study did not include long-term clinical endpoints, such as definitive wound healing time, infection rates, or flap success rates. While these metrics are essential for evaluating individual clinical outcomes, the primary focus of this research was to provide a macroscopic analysis of the systemic resource gaps associated with inter-hospital transfers between tertiary centers. By quantifying the intensity of required interventions—such as the number of operative debridements and specialized wound care sessions—we sought to highlight the often-overlooked reconstructive burden within the regional trauma care system. Future research should aim to include multiple institutions to provide a more comprehensive understanding of wound management practices and their impact on interhospital transfers. Such studies could help to identify common challenges and best practices across different healthcare settings.

In conclusion, this study reaffirms the critical importance of effective wound management in trauma care. Our findings highlight a significant clinical gap in the current trauma system, showcasing a clear need for a ‘bridge’ between trauma services and PS specialized care. The high frequency of inter-hospital transfers between tertiary centers suggests that even well-equipped facilities face challenges in providing the intensive reconstructive resources required for complex wounds. By ensuring timely and specialized interventions by plastic surgeons, regional trauma centers can significantly enhance the quality of care and reduce unnecessary transfers. This study calls for a renewed focus on wound management as a central component of trauma care, advocating for the integration of plastic surgery into regional trauma systems and increased resources dedicated to this vital aspect of patient treatment.

## Conclusions

This study identifies a significant clinical and systemic gap within the current trauma care continuum, specifically regarding the management of complex soft tissue injuries. Our findings demonstrate that a substantial proportion of inter-hospital transfers between tertiary centers is significantly associated with unresolved reconstructive needs, which often persist despite successful initial stabilization. Rather than representing a failure of management, these transfers may reflect a proactive triage strategy for optimal care delivery. This suggests that current trauma protocols, while highly effective for vital sign stabilization and skeletal fixation, may lack a standardized strategic framework for concurrent specialized wound care in high-severity cases, such as comminuted fractures. To optimize regional trauma systems, it is essential to integrate a proactive, multidisciplinary consultation model with plastic surgery departments from the early stages of care. Ultimately, integrating plastic surgery into trauma systems is key to optimizing resource utilization and enhancing the overall effectiveness of regional trauma care.

## Data Availability

Data supporting the findings of this study are available from the corresponding author upon reasonable request. We support the principles of open science and are pleased to provide data to facilitate further research and academic collaboration in this field.
